# Designing equitable and effective partnerships for transformative health outcomes in Port Loko District, Sierra Leone: a grassroots public health perspective

**DOI:** 10.1093/inthealth/ihaf159

**Published:** 2026-02-14

**Authors:** Morlai Sesay

**Affiliations:** School of Public Health, Southern Medical University, 1023-1063 Shatai Nan Road, Guangzhou 510515, China

**Keywords:** community health workers, data democratization, equitable and effective partnerships, grassroots health, health systems strengthening, Sierra Leone

## Abstract

Port Loko District, Sierra Leone, has for a long time suffered from abject health inequalities fuelled by top-down, externally imposed health interventions without the support of community participation and sustainable institutions. My field experience shows that equitable and effective partnerships based on the five pillars of participatory co-design, mutual capacity development, democratization of information, resource allocation with equity prioritization and ongoing feedback with cultural humility hold the key to revolutionary health outcomes. Health alliances should solidify co-leadership by communities in leadership, invest in local data systems and tie funding with open, equity-driven processes to ensure sustainable gains in health.

## Introduction

This article draws on my 4 y of frontline implementation experience (2019–2023) to propose a practical framework for reconfiguring global health partnerships to produce sustainable, equity-focused outcomes in low-resource settings. Shifting from extractive to collaborative models is not only an ethical imperative but also a pragmatic route to better health indicators. A review by Price et al.^1^ and case analyses from Liberia^2^ show that equitable partnerships, defined as shared governance, mutual capacity strengthening and transparent resource flows, are a determinant of progress toward universal health coverage. This article contributes granular, longitudinal evidence from Sierra Leone that corroborates and extends those findings.

Port Loko District in the North-West region of Sierra Leone is a quintessential example of issues around achieving health equity in low-resource settings. The country suffers from extreme and persistent healthcare challenges, ranging from a high rate of maternal mortality (717 per 100 000 live births) and a newborn mortality rate of 31 deaths per 1000 live births, to excessive infectious disease burdens and poor infrastructure and donor-driven agendas.^3^ The traditional global health collaborations reinforce colonial hierarchical orders under which foreign institutions occupy decision-making spaces while local stakeholders remain relegated to implementers.^4^

**Figure 1.**
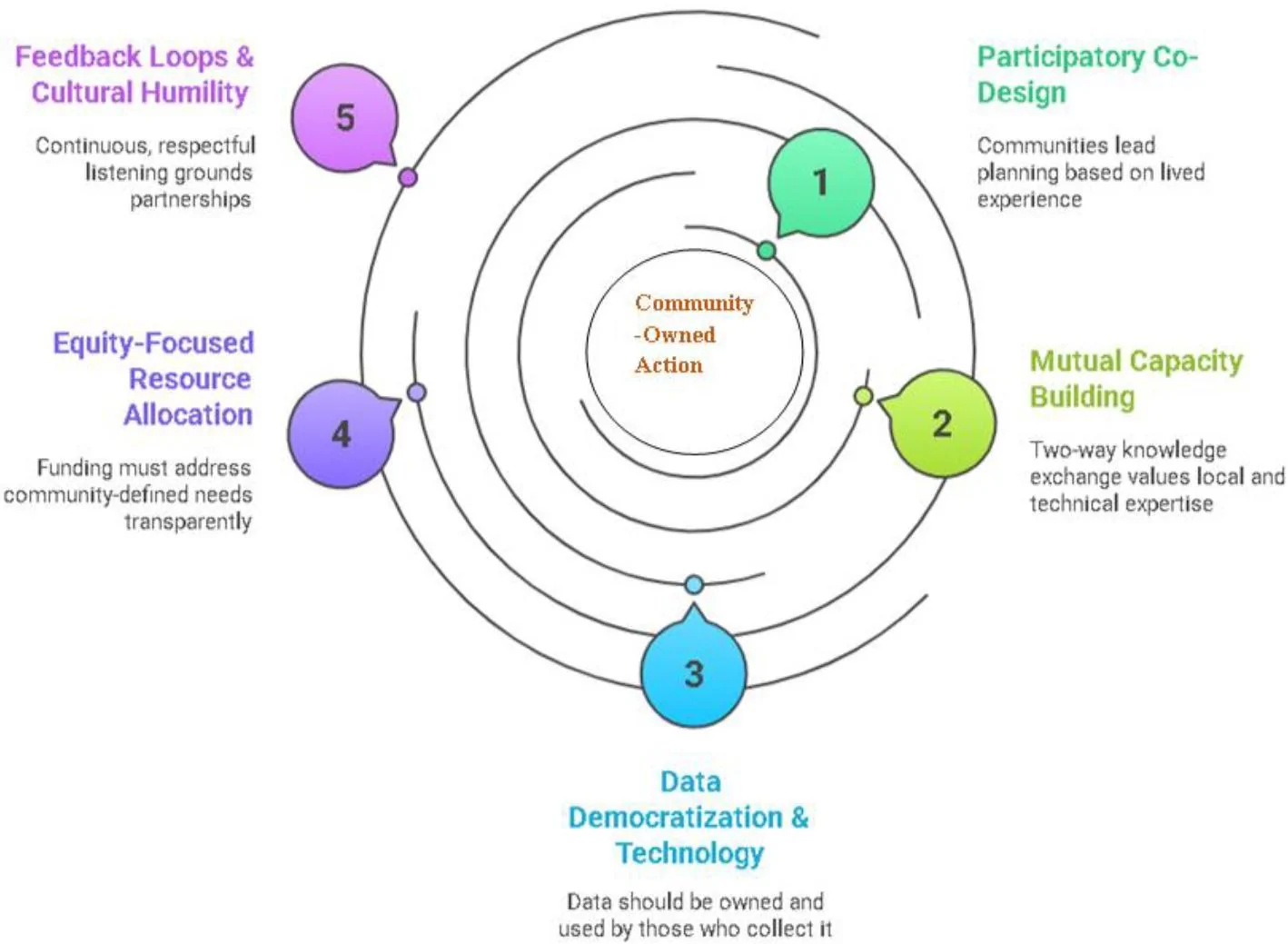
A framework for equitable and effective health partnerships in Port Loko District.

Post-Ebola reconstruction left Port Loko with 0.8 doctors per 10 000 population and 50% of facilities without electricity. Between 2019 and 2023, the district received seven vertical programs (Global Fund/Global Fund to Fight AIDS, Tuberculosis, and Malaria; U.S. President’s Malaria Initiative; Global Alliance for Vaccines and Immunisation; Catholic Relief Services; COVID-19 response; World Bank Community Emergency Response Program; and Irish Aid malaria grant), each with separate log-frames and reporting lines. Community health workers (CHWs) sometimes remained unpaid or received half payment and district leadership was routinely informed of budget allocations only after commitments had been signed in Freetown or Geneva. In this context, I argue, Port Loko is a ‘quintessential example’ of how global health partnerships can perpetuate inequity; the remainder of the article unpacks concrete episodes from this setting to illustrate the five-pillar alternative.

## Methods: context and field experience

This assessment is based on my actual practice of implementation from 2019 to 2023 in Port Loko District in the following positions: CHW focal person (2020–2023), contact tracer supervisor (June–August 2021), COVID-19 case investigator (November 2020–April 2021), malaria supervisor (2021–2022) and district logistics officer (2019). In all these roles I worked closely with the district health management team, traditional leaders, women’s groups and CHWs and witnessed the demoralizing effect of top-down approaches of repeatedly bypassing local knowledge and the transformative power of community-driven alliances. The lessons learned stemmed from a reflexive analysis of the experience.

### Lessons learned: a five-pillar framework for equitable and effective partnerships

#### Participatory co-design of health programs

Top-down programmatic designs frequently fail to address on-the-ground realities. During measles campaigns, for instance, logistical decisions by central authorities often neglected last-mile delivery challenges in remote Port Loko communities. As the CHW focal person, I led community planning meetings in which villagers mapped local health priorities. Their input into mobile clinic scheduling resulted in a 30% increase in vaccination uptake. Tools like community scorecards and participatory rural appraisals institutionalize this process. Before launching a measles campaign, discussions with youth networks and women’s groups ensured that the messaging was culturally appropriate and that logistics were community-driven.

#### Mutual capacity building

Global health can view capacity building as a one-way activity where locals are trained but existing knowledge is overlooked. Locals, however, have valuable indigenous knowledge, contextual expertise and trust networks. By piloting the Community Health Information System (CHIS) tool, the inclusion of Ministry of Health trainers alongside CHWs resulted in user-initiated changes that improved data quality and utilisation of the system. The mutualism, a situation where the technical partners offer training while they learn from the locals’ expertise, is key to their sustainability.

#### Data democratization and use of technology

Digital technologies have the potential to revolutionize health systems but need to be deployed inclusively. In many rural settings, and certainly in the villages I worked in, health information is either not collected in real time or is shared only in formats that communities cannot easily interpret or act upon. Models such as community-led monitoring (where users themselves collect, analyse and feed data back into action) have shown promise elsewhere, yet during 2020–23, Port Loko had no systematic community-led monitoring mechanism; the CHIS and electronic Integrated Disease Surveillance and Response (eIDSR) system pilots described below were designed to fill that gap. In the response to COVID-19, data generated by contact tracing was transmitted to the national level but only infrequently cycled back to district teams, slowing response. We trained CHWs under the Community-Led Action program to report online in real time through their health facilities’ eIDSR systems, enabling the district health management team (DHMT) to redeploy testing kits and personal protective equipment effectively. Visualization of data through dashboards at the community level created key local agency and ownership.

#### Equity-focused resource allocation

Resources tend to correspond to donor interests instead of needs as identified by the community. In 2021, one malaria grant covered bed net distribution but not stipends for CHWs, thereby undermining implementation in distant villages. By distributing DHMT-initiated budget briefs among district partners, we managed to lobby for transportation allowances and logistical support to reach distant villages. Co-authoring grants and transparency in budgets help ensure equal distribution of resources.

#### Feedback mechanisms and cultural humility

Effective partnerships require continuous listening. At a maternal health session, participants misunderstood key messages due to language barriers.^5^ Without feedback mechanisms such miscommunications persist. We instituted monthly ‘listening circles’ where women discussed programs in Krio and Temne, leading to culturally appropriate revisions like visual aids for low-literacy groups. Cultural humility, where global actors challenge their assumptions, is equally vital. During my DHMT internship, I observed communities rejecting culturally insensitive interventions that disregarded traditional healing practices (Figure 1).

## Discussion

The five-pillar approach represents a paradigm shift from extractive to collaborative global health practice. In my experience in the Port Loko District, the people most respected by communities—district-level nurses, midwives and veteran CHWs—are frequently the least empowered within national governance structures. The response to the COVID-19 pandemic illustrated the pitfalls of top-down mandates as well as the success of localized initiatives like quarantine measures led by the villages. By bridging indigenous leadership with the right technology, such as CHIS and eIDSR, rapid culturally suitable responses became possible.

It is challenging to put this model into practice, given the donor’s resistance to relinquishing control, institutional resistance and funding frameworks oriented toward measurable short-term results rather than sustainable district capacity. However, the Port Loko lessons demonstrate that the obstacles may be surmounted by continued advocacy and demonstration of better results.

## Conclusions and recommendations

The development of balanced partnerships is an ethical need as well as a strategic imperative for sustained improvement in health. As I noted in my field diary, investing in community-owned activities to drive interventions is reflective, resilient and sustainable. The following five measures should be implemented in the order presented; each builds on the previous one to move from power-sharing to measurable equity impact.

1. Include the co-leadership of health communities in health governance architectures and partnership agreements.
2. Invest in local data systems and open-source technologies owned and operated by communities.
3. Tie funding to equity measures and transparent processes that prioritize community-defined needs.
4. Organize mechanisms for receiving feedback based on cultural humility and respect for country expertise.
5. Recognize and reward CHWs as valued partners and not as voluntary add-ons.

The evolution of ‘power sharing → equity → impact’ will have the powerful benefit of transformation not only for the Port Loko District but also for all global health equity-seeking communities.

## Data Availability

All data are available in the article.
